# Algal Symbionts Indicate Heatwave Vulnerability in Corals From Hotspots but Not From Thermal Refugia

**DOI:** 10.1111/mec.70243

**Published:** 2026-01-17

**Authors:** Daisy Buzzoni, Liam Lachs, Elizabeth Beauchamp, Leah Bukurou, John Bythell, Alasdair J. Edwards, Yimnang Golbuu, Adriana Humanes, Helios M. Martinez, Geory Mereb, Julia K. Baum, James R. Guest

**Affiliations:** ^1^ Department of Biology University of Victoria Victoria British Columbia Canada; ^2^ School of Natural and Environmental Sciences Newcastle University Newcastle upon Tyne UK; ^3^ Palau International Coral Reef Center Koror Palau; ^4^ The Nature Conservancy, Micronesia and Polynesia Koror Palau

**Keywords:** coral bleaching, degree heating weeks, DNA metabarcoding, marine heatwaves, Symbiodiniaceae, thermal refugia

## Abstract

Reef‐building corals face continued declines due to climate change‐amplified marine heatwaves. In addition to affecting coral heat tolerance, corals' algal endosymbionts (family Symbiodiniaceae) can reflect their prior heatwave exposure, although understanding is often limited to heatwave‐induced shifts between symbiont genera. Here, we used ITS2 metabarcoding to characterise Symbiodiniaceae assemblages in 293 individuals of the common Indo‐Pacific coral *Acropora aff. digitifera* in Palau (Western Pacific), between two outer‐reef regions with contrasting heatwave histories. During the strongest recorded heatwaves, southwestern ‘hotspot’ reefs have typically accrued an additional 2°C‐weeks of heat stress compared to thermal ‘refugia’ located 60 km north. In contrast to previous studies that observed declines in symbiont richness following heat stress, we found a greater diversity of symbiont taxa and low‐abundance sequence variants in ‘hotspot’ corals, predominantly within the C40 lineage in genus *Cladocopium*. Combining these data with experimental heatwave performance from 168 of these corals revealed that approximately 10% of heat tolerance variability at hotspot reefs was associated with hosting different symbiont taxa. Compared to other hotspot corals, those hosting symbionts with the C15h sequence variant suffered bleaching mortality at 0.8°C‐weeks lower heat stress. Despite higher variability in heat tolerance among corals from thermal refugia compared to hotspot reefs, we found no association between heat tolerance and the symbionts hosted by refugium corals. As the world's coral reefs are exposed to intensifying marine heatwaves under accelerating climate change, the low‐abundance variants that characterise symbionts within genera or lineages may become increasingly important indicators of poor heatwave tolerance.

## 1 Introduction

Marine heatwaves are restructuring populations, imposing directional selection through mass‐mortality and favoring specific genotypes whilst simultaneously eroding standing genetic and phenotypic variation (Coleman and Wernberg 2020; Gurgel et al. 2020; Burgess et al. 2021; Starko et al. 2023; Lachs, Bozec, et al. 2024). Reef‐building corals (order Scleractinia) are especially vulnerable to the effects of marine heatwaves, since they exist in obligate symbioses that are highly sensitive to environmental disturbance (Glynn 1996) and are often distributed close to their thermal tolerance limit (Smith et al. 2023). Some of the variation in coral heat tolerance over scales of 300–1300 km (across latitudinal gradients of the Great Barrier Reef for example) has been attributed to coral adaptation or acclimatisation to differential historical heat stress (Guest et al. 2012; Denis et al. 2024; Naugle et al. 2024). However, the effects of marine heatwaves on coral heat tolerance are also influenced by the timing and the spatial extent of prior exposure (Hughes et al. 2019; Marzonie et al. 2022). The effects of historical heatwave exposure on coral thermal tolerance across the smaller spatial scales most relevant for reef‐management (< 100 km) have been rarely considered (Lachs, Humanes, et al. 2024; Naugle et al. 2024), with more focus on the influence of environmental variability at these small spatial scales (van Woesik et al. 2012; Voolstra et al. 2020; Brown et al. 2024). Heatwave tolerance may increase in coral populations through adaptation to frequent heat stress (Lachs, Bozec, et al. 2024), although reefs that experience consistently less heatwave stress, which we refer to here as thermal ‘refugia’, may represent crucial reservoirs of genetic variation and adaptive potential as heatwaves force population bottlenecks in surrounding habitat (Keppel et al. 2012; Leiva et al. 2025). As marine heatwaves continue to increase in severity, duration and frequency under accelerating climate change (Oliver et al. 2021), few reefs are expected to escape regular heat stress in the coming decades (McWhorter et al. 2022).

Environmental variation can drive local adaptation even in populations with high gene flow and as such population structure can persist despite long‐range dispersal of coral larvae (Richardson et al. 2014). The reef‐building coral *Acropora digitifera* has shown high population connectivity between islands of the tropical Pacific over 1000 km apart and has the capacity for extensive dispersal (Davies et al. 2015). Despite high connectivity, considerable variation in *A. aff. digitifera* heatwave tolerance, with a substantial heritable coral genetic basis, is evident between nearby reefs (< 100 km) and even within reefs (< 100 m) (Humanes et al. 2022; Lachs, Humanes, et al. 2024). The north of Palau's barrier reef system has consistently been exposed to milder heat stress conditions during the most intense marine heatwaves (van Woesik et al. 2012; Lachs, Humanes, et al. 2024), potentially owing to frequent flushing of northern lagoons through reef channels compared to higher tidal retention surrounding southwestern reefs (Golbuu et al. 2012, 2016). This marked local variation in historical heat stress between Palau's northern thermal ‘refugia’ and southwestern ‘hotspots’, delineated recently from satellite data (Lachs, Humanes, et al. 2024), is associated with corresponding genetic divergence in Palauan populations of several reef‐building corals (Cros et al. 2016; Rivera et al. 2022).

Phenotypic variation within coral populations can reflect differences in microbial holobiont composition, with corals’ associated microalgal endosymbionts (Family Symbiodiniaceae) capable of generating substantial variation in thermal tolerance (Berkelmans and van Oppen 2006; Silverstein et al. 2015). Many species in the genus *Acropora* exhibit flexibility in their associations with different symbiont genera, which is characteristic of the reduced symbiont specificity attributed to corals that acquire their symbionts from the environment rather than inheriting them from a parent (Fabina et al. 2012; Turnham et al. 2021). However, *Acropora* spp. have shown high fidelity to regionally‐specific lineages within *Cladocopium* (Thomas et al. 2014; Zarate et al. 2024), the most phylogenetically and phenotypically diverse genus within Symbiodiniaceae (LaJeunesse et al. 2018). Palau's *A. aff. digitifera* population has been found to host symbionts in genera *Durusdinium* and *Symbiodinium* (Humanes et al. 2024), but its associations with *Cladocopium* are almost exclusively with the ‘C40’ lineage, formally described as *Cladocopium madreporum* (Davies et al. 2020; Humanes et al. 2022; Butler et al. 2023; Lachs et al. 2023; Lewis et al. 2024). Despite this C40‐specificity, spatial structuring of symbionts within a single *Cladocopium* lineage has been detected within other highly connected *Acropora* spp. host populations (Davies et al. 2020; Kriefall et al. 2022; Armstrong et al. 2024; Naugle et al. 2024), and therefore may also exist between Palau's *A. aff. digitifera* population and different C40 symbiont taxa.

Exposure to heat stress has been linked to reductions in the diversity of Symbiodiniaceae in reef‐building coral hosts. The erosion of symbiont diversity with heat stress can reflect the replacement of heat‐sensitive *Cladocopium* symbionts by less taxonomically diverse, stress‐tolerant genera *Durusdinium* or *Symbiodinium* (Pettay et al. 2015; Claar et al. 2020; Jain et al. 2020; Quigley et al. 2022). The loss of symbiont richness from within a single *Cladocopium* lineage has also been documented in some coral populations following heat stress (Kriefall et al. 2022; Starko et al. 2023; Leiva et al. 2025). Therefore, local thermal refugia that have avoided such losses may prove important reservoirs for symbiotic diversity at this fine taxonomic scale.

Contrary to expectations of increased heat tolerance at hotspot reefs in Palau, owing to coral adaptation or acclimatisation with higher exposure to historical heat stress, Lachs, Humanes, et al. (2024) observed reduced heat tolerance of *Acropora aff. digitifera* from hotspots, compared to their northern refugium‐sourced conspecifics which also exhibited greater variation in heat tolerance. Here, we investigate whether differences in historical heat stress in this reef system are associated with the Symbiodiniaceae taxa hosted by *Acropora aff. digitifera*, with corresponding links to coral heat tolerance. We hypothesise that (i) corals from hotspot reefs host less diverse Symbiodiniaceae assemblages due to the loss of symbiont richness with historical heat stress, and (ii) variation in heat tolerance between corals from reefs that have experienced comparatively less historical heat stress corresponds to the Symbiodiniaceae taxa they host. This study provides insights into associations between symbionts and historical heat stress regimes beyond shifts among Symbiodiniaceae genera. Our results also contribute to a burgeoning appreciation of symbiont variation at fine taxonomic scales within *Cladocopium* and corresponding links to heat tolerance within coral populations.

## 2 Materials and Methods

### 2.1 Historical Thermal Regimes

**FIGURE 1:**
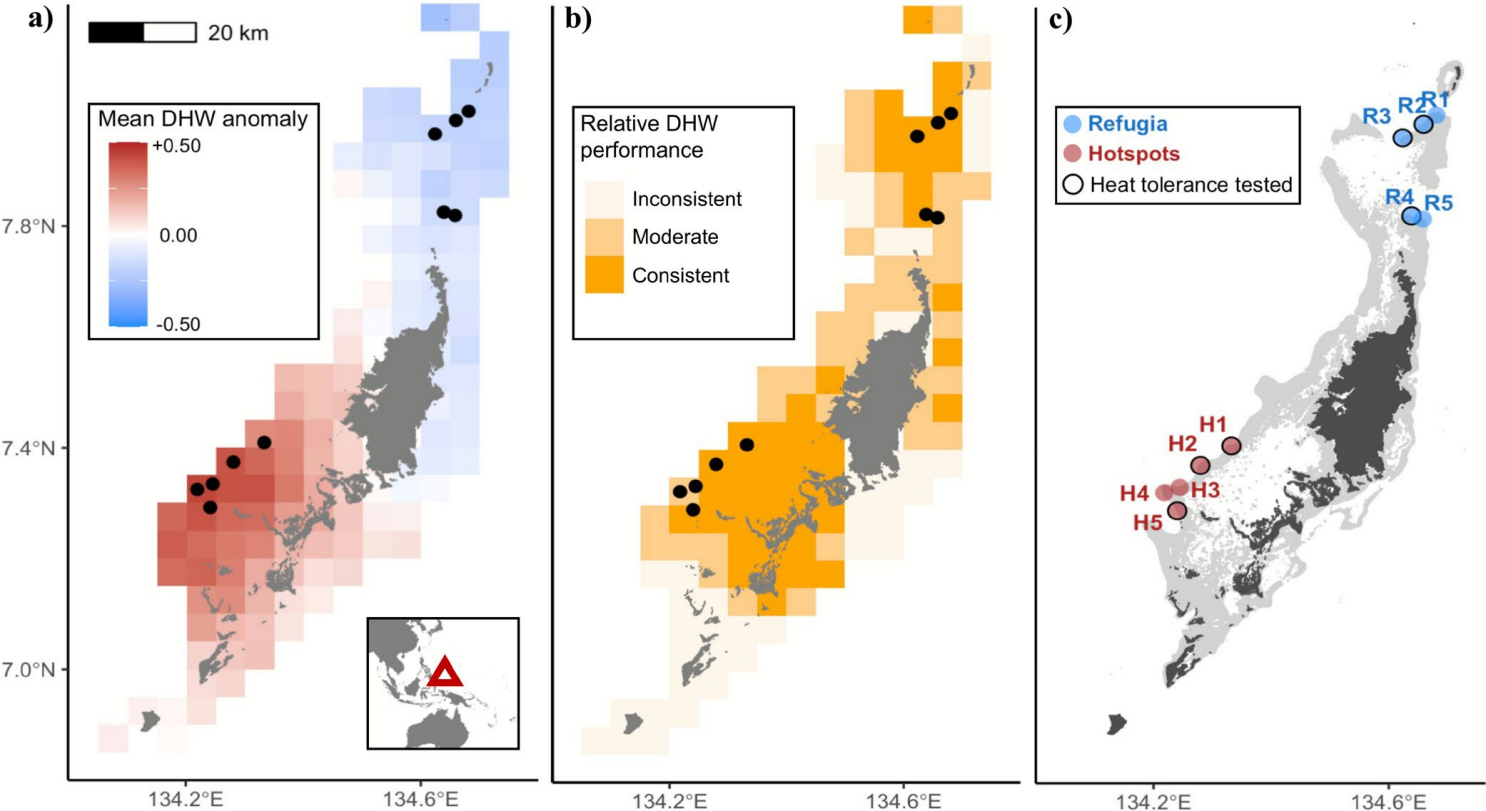
Sampling across Palau's gradient of historical heat stress, modified from Lachs, Humanes, et al. (2024). (a) Coral collection sites are shown in black, against a map of reef pixels. Pixels are coloured according to mean annual Degree Heating Week anomaly between 1985 and 2020 above or below the average across Palau's reefs. (b) Pixel shading indicates standard deviation of annual percentile rankings of reef pixels, such that lower values (darker shading) correspond to higher consistency in DHW ranking. (c) Locations of the five thermal ‘Refugium’ reefs (R1–R5) and the five thermal ‘Hotspots’ (H1–H5) where *Acropora aff. digitifera* branches were collected. Replicate branches from three of the five sites in each region were subjected to an experimental heatwave in addition to symbiont characterisation (sites outlined in black). Dark grey areas represent land and light grey indicates reef extent (extracted from; Anderson 2007).

The coral individuals investigated in this study originated from five replicate sites from thermal refugia and hotspot reefs. Three of the replicate sites in each region (sites R2, R3, R4, H1, H2 and H5) correspond to the sites and corals used in Lachs, Humanes, et al. (2024) that were subjected to an experimental heatwave (see Section 2.2), as previously reported in Lachs, Humanes, et al. (2024). Corals from the two additional replicate sites in each region were sampled solely for symbiont characterisation and were not included in the experimental heatwave. The characterisation of historic thermal stress regimes, given in full detail in Lachs, Humanes, et al. (2024), was based on 0.05° by 0.05° latitude‐longitude resolution maps of annual peak cumulative heat stress for the period 1985–2020. Heat stress was calculated in Degree Heating Weeks (DHW, in units of °C‐weeks)– a metric that incorporates both the duration and intensity of heat stress, such that > 8°C‐weeks is strongly associated with reef‐wide coral bleaching (Heron et al. 2016; Lachs et al. 2021). By mapping the annual heat stress reefs received relative to one another (DHW anomaly), and then calculating the inter‐annual mean DHW anomaly and the inter‐annual consistency with which reefs were ranked (in terms of the mean DHW percentile across years), Lachs, Humanes, et al. (2024) delineated persistent hotspots and thermal refugia, as reefs that consistently experience higher‐than‐average heat stress and lower‐than‐average heat stress, respectively (Figure 1).

### 2.2 Coral Collection and Experimental Heatwave Exposure

*Acropora aff. digitifera* corals have a corymbose morphology and are widely distributed across Indo‐Pacific high wave‐energy reefs. Fore reefs between 3 and 8 m below sea level were chosen as collection sites within target thermal hotspot or refugium pixels. At each site, transects were laid along a 5–6 m isobath and *A. aff. digitifera* corals with at least 20 basal branches (approximately 20 cm in diameter) and at least 5 m apart were selected, to reduce the probability of selecting clones (Baums et al. 2019). Corals were selected based on their morphological affinity to the nominal species *Acropora digitifera* , hence the ‘*aff*.’ qualifier is used here. From each coral, branches (6 for heat stress evaluation and 1 for DNA sequencing) were broken off at their bases and stored in seawater coolers until they were transferred to experimental tanks at the Palau International Coral Reef Center (Koror, Palau) later the same day.

Coral branches were allowed to acclimate to conditions in a series of 40 L flow‐through race‐way tanks for 7–10 days following collection in April 2022. Five branches per coral were transferred to randomised positions across 10 experimental tanks and the sixth to one of two control tanks. After acclimation, experimental tank temperatures were increased by 0.4°C on Day 1 and by 0.5°C on Days 3, 5, 7, and 13 to reach a final temperature of approximately 32.5°C. This temperature was maintained for over 5 weeks until all branches had died, accumulating up to 16°C‐weeks. One branch in the control treatment suffered mortality and, as such, all experimental branches originating from this coral were removed from subsequent analyses (Table S1). Further details of this experimental heatwave exposure are given in Lachs, Humanes, et al. (2024).

The visible progression of coral bleaching, whilst a valuable indicator of coral health and symbiotic state, does not necessarily predict stress‐induced coral mortality (Hughes et al. 2019; Matsuda et al. 2020). To capture both responses, Lachs, Humanes, et al. (2024) recorded the visual progression of both bleaching and mortality in experimental fragments and calculated a bleaching and mortality index (from 0 to 1) for each coral as heat stress advanced. This facilitated the calculation of the 50% effective dose (ED_50_) for heat stress, DHW_50_, the accumulated heat stress at which a coral's bleaching and mortality index reaches 0.5 (equivalent to all replicate branches being bleached), used here as an ecologically meaningful measure of tolerance to simulated long‐term marine heatwave stress.

### 2.3 Symbiodiniaceae DNA Extraction and Sequencing

Coral branches designated for sequencing were wrapped in aluminium foil and stored at −80°C by the end of the collection day upon removal from a seawater cooler. Each wrapped branch was flash‐frozen in liquid nitrogen and crushed into a fine powder using a sterilised pollen press and hammer. The powdered branch was briefly mixed with a spatula and ~2 mm^3^ of sample was retained for DNA extraction using a DNeasy Blood and Tissue kit (Qiagen). The manufacturer's protocol was modified to include an overnight lysis, and samples were carefully inverted rather than vortexed prior to being passed through spin columns to avoid shearing of DNA by skeletal fragments (Baums and Kitchen 2020). In addition, the supernatant of lysed samples was transferred into new microcentrifuge tubes to remove large skeletal fragments, and then again following 1 min at 6000 rpm in a centrifuge to remove smaller skeletal fragments, and two 200 μL elutions were performed and combined to maximise final DNA yield. The internal transcribed spacer 2 (ITS2) ribosomal region was amplified using SYM_VAR primer pairs (5′CAGCTTCTGGACGTTGYGTTGG3′; 5′CGGGTTCWCTTGTYTGACTTCATGC3′) (Hume et al. 2013, 2018) and combined to form unique paired indexes for sample demultiplexing. A no‐template control from each 96‐well amplification and a total of three negative extraction controls were used to check for contamination using gel electrophoresis. Amplicon purification and normalisation was performed using Just‐a‐plate 96 PCR Normalisation and Purification kits (Charm Biotech). The resulting PCR products were pooled and quantified with Qubit 3.0 fluorometry (Thermo Fisher Scientific). Amplicon sequence data were generated using Illumina MiSeq v2 (Illumina Inc.) performed at the John G. Shedd Aquarium (Chicago) Molecular Ecology Laboratory, generating paired‐end 250 base‐pairs and yielding on average 25,154 raw contigs per sample.

### 2.4 Bioinformatics & Classifying Symbiodiniaceae Sequence Data

ITS2 sequence data were analysed using two workflows to classify distinct sequence variants: (i) SymPortal, which uses minimum entropy decomposition to identify defining intragenomic variants (DIVs) and uses co‐occurrence patterns to further cluster these into ITS2 profiles, representing putative species (Hume et al. 2019) and (ii) DADA2, which identifies amplicon sequence variants (ASVs) using a denoising algorithm that models sequencing errors (Callahan et al. 2016). These two bioinformatics pipelines represent different approaches to resolving intra‐ and inter‐genomic sequence variants from the multi‐copy ITS2 region in Symbiodiniaceae, hence combining these methods allows for more robust interpretation of sequencing results (Quigley et al. 2022; Davies et al. 2023). The DADA2 approach offers greater sensitivity in differentiating sequence variants, whilst the SymPortal approach aims to distinguish most intra‐ and inter‐genomic variation, although ASVs and DIVs cannot confidently be confirmed as intra‐ or inter‐genomic from these data and are therefore referred to as sequence variants. Sequence data were submitted to the remotely hosted SymPortal database. For the DADA2 pipeline, primer sequences were removed using the ‘cutadapt’ tool (Martin 2011), then forward and reverse reads were denoised and merged using default parameters (truncating reads at the first instance the quality score falls below 2 and permitting a maximum of 2 expected errors in each forward or reverse read). To minimise the effect of uneven sample sequencing depths on the detection of low abundance variants, sample coverage of ASV and DIV richness was modelled against sequencing depth using the ‘iNEXT’ package (Hsieh et al. 2024), revealing estimated sample coverage greater than 0.999 at 1000 reads (Figure S1). Hence, 6 samples with < 1000 total post‐processing Symbiodiniaceae reads were removed from analyses, including 5 samples from corals in the experimental heatwave, leaving data from 293 corals (168 of which were in the experimental heatwave) retained for analysis (Table S1).

SymPortal sequence data yielded a mean of 10,587 post‐processing Symbiodiniaceae reads per sample from a total of 61 distinct DIVs which were further collapsed into 23 distinct ITS2 profiles. A mean of 9996 Symbiodiniaceae reads per sample was retained from the DADA2 output for a total of 92 distinct ASVs. The most numerically abundant (the highest relative read abundance) ITS2 profile in each sample comprised on average 99.7% (over 75% in all cases) of Symbiodiniaceae reads, the dominant profile. Out of 293 samples, 13 contained an additional background ITS2 profile, comprising on average 7.9% of Symbiodiniaceae reads. Where ITS2 profiles are displayed, a colour scale has been assigned based on profiles’ average defining proportions of sequence variants (Table S2, Figure S2). All ITS2 profiles, DIVs and ASVs detected were within genus *Cladocopium*, with DIVs and ITS2 profile taxonomies assigned by using reference sequences within the SymPortal community database. Unique ASVs were numbered and assigned to the genus level within DADA2 using a naïve Bayesian classifier algorithm (Wang et al. 2007) with a minimum bootstrap confidence of 80 using a comprehensive Symbiodiniaceae reference database available within the online data repository (Franklin et al. 2012; Bongaerts et al. 2013; Nitschke 2020). Subsequently, ASV ancestral *Cladocopium* lineages were assigned based on their closest match within the same reference database. ASV and DIV reference sequences were aligned using the ‘MUSCLE’ algorithm implemented in the ‘msa’ R‐package (Edgar 2004; Bonatesta et al. 2024). Pairwise distance calculations were performed on aligned sequences in ‘seqinr’ (Charif 2023), and these were used to construct neighbour‐joining trees of ASVs and DIVs using the ‘ape’ R package (Paradis 2024). Pairwise distances between ITS2 profiles for the construction of a neighbour‐joining tree were provided by SymPortal (Figure S3).

### 2.5 Statistical Analyses

Indicator species analyses were performed using multi‐level pattern analyses on symbiont assemblages followed by permutation testing (De Cáceres and Legendre 2009; De Cáceres et al. 2010). The ‘Indicator Value’ method was used to detect symbiont ASVs or DIVs that, when present, were significant predictors of a coral's hotspot or refugium origin, based on both the regional specificity and regional occurrences of sequence variants. To compare the heterogeneity of symbionts hosted by corals collected from hotspot reefs and from refugia, both alpha diversity (diversity within coral samples) and beta dispersion (symbiont assemblage dissimilarity between samples) were assessed. Alpha diversity of ITS2 profiles within each coral was estimated using the phylogenetic‐Hill richness metric, which incorporates profile richness and phylogenetic spread by integrating the sum of neighbour‐joining tree branch lengths (Figure S3) connecting the profiles in each sample (Li 2023). Dissimilarities between symbiont assemblages were calculated as weighted unifrac distances (sequence dissimilarity between composite DIV or ASV variants) and ordinated. The beta dispersion between hotspot samples and between refugium samples was compared based on sample distances to centroids from weighted unifrac ordinations.

The Bartlett test for homogeneity of variances (around group means) was used to compare the variances in coral heat tolerance (DHW_50_) between regions. Indicator species analyses of heat tolerance were performed separately on all ASVs, DIVs and ITS2 profiles to detect symbiont indicator candidates: a point‐biserial correlation method was used to test symbiont associations with corals in a variety of heat tolerance quantiles (e.g., tertiles, quartiles and pentiles). Only symbiont taxa or sequence variants identified as indicator candidates for heat tolerance were assessed further. The strength of the relationships between coral DHW_50_ and symbiont indicator candidates’ relative read abundance (RRA) within samples was formally assessed using generalised linear mixed‐effects models with Gamma error distributions (to relax the assumption of homoscedasticity). Collection site was included as a random effect in linear mixed‐effects models and four putative outlier points (2 hotspot corals and 2 refugium corals) were excluded due to their studentised residuals exceeding ±3 (Fox and Weisberg 2018). Differences in the relative read abundances of sequence variants between samples comprising the same ITS2 profile may reflect sequencing biases rather than real differences in variant relative abundances (Hume et al. 2019). Hence, linear models of heat tolerance were constructed using the RRA of a variant in each sample (‘sample RRA’ models) in case of erroneous clustering of distinct taxa into ITS2 profiles and analogous ‘mean profile RRA’ models were created using the mean RRA of the variant across samples hosting the same ITS2 profile(s). Figure S4 shows the variability in sample RRA that was collapsed into mean profile RRA for four variants of interest. The variance in DHW_50_ explained by symbiont fixed effects was estimated as the marginal *R* ^2^ of linear mixed‐effects models (Nakagawa and Schielzeth 2013). Post hoc Tukey's pairwise tests were used to test for significant between‐group differences in mean DHW_50_ according to the dominant ITS2 profile hosted.

## 3 Results

### 3.1 Hotspot Corals Host a Greater Diversity of Symbiont Species

**FIGURE 2:**
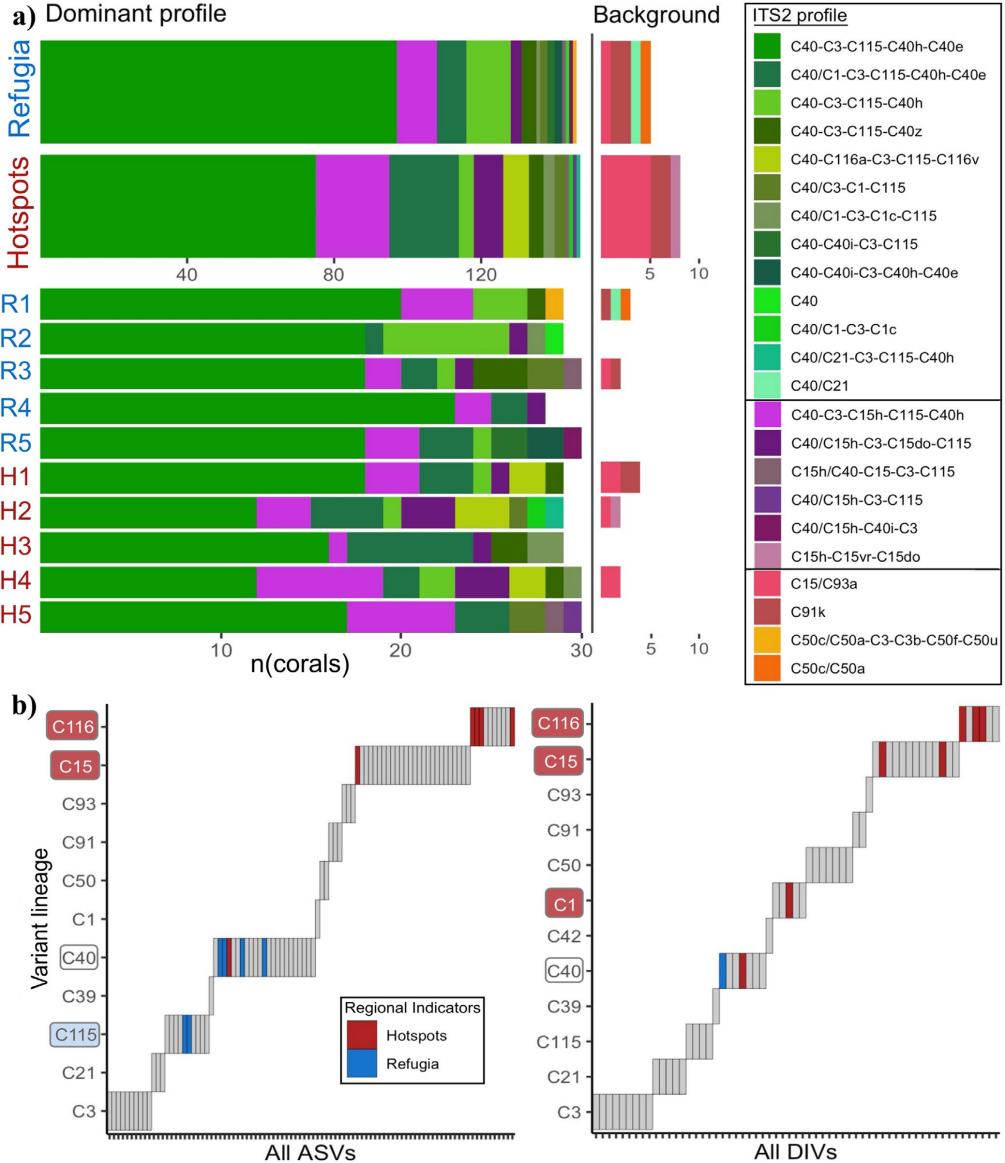
Hotspot and refugium symbionts characterised by their low‐abundance variants. (a) Number of corals hosting each *Cladocopium* ITS2 profile from hotspot reefs (*n* = 147, H1–H5) and thermal refugia (*n* = 146, R1–R5). The dominant profile and additional background profile (if present, comprising < 10% of sample symbiont reads) are shown. Unique ITS2 profiles are represented by a colour scale, grouped in the figure legend by those comprising mainly the C40 variant (top, green hues, C40 defining sequence proportions ≥ 0.5) and those containing the C15h variant (middle, purple hues), with additional profiles grouped below. Profiles are ordered within each group by decreasing occurrence in the dataset in the figure legend. (b) Results of presence/absence indicator analysis, with symbiont sequence variants (ASVs or DIVs) represented by *x*‐axis ticks (ordered by lineage). Colours represent sequence variants that are significant indicators of refugia (blue) or hotspot (red) reefs, otherwise variants are shown in grey. Lineages containing indicator variants are surrounded by borders and those containing only hotspot or only refugium indicators are shaded red or blue respectively.

*Cladocopium* C40‐C3‐C115‐C40h‐C40e was the most common profile detected in *A. aff. digitifera* corals sampled across all 10 sites, yet this was the dominant profile in only 51% of hotspot corals compared to 67% of refugium corals (Figure 2a). The six profiles containing the C15h sequence variant were more common in hotspot corals, detected in 21% of individuals compared to only 11% of refugium corals, largely driven by profiles C40‐C3‐C15h‐C115‐C40h and C40/C15h‐C3‐C15do‐C115 (in which C15h is present at defining proportions of 0.05 and 0.38 respectively, Table S2). Other region‐specific symbiont distributions include the detection of C116 variants in the C40‐C116a‐C3‐C115‐C116v profile in seven corals exclusively at hotspot reef sites, and profiles containing C50 variants in two refugium corals. Indicator analyses revealed sequence variants deemed to indicate a coral's hotspot or refugium origin, when present: six ASVs and one DIV were significant refugium indicators, compared to six ASVs and seven DIV indicators of hotspot corals (Figure 2b, indicator value test statistics given in Table S3). Across both ASV and DIV bioinformatics methods, the presence of several C15 and C116 variants was significantly associated with corals originating from hotspot reefs, in accordance with increased occurrences of C15‐ and C116‐containing ITS2 profiles across hotspot corals (Figure 2a). All C15 and C116 variants were found either in background profiles (as C15h‐C15vr‐C15do or C15/C93a hosted alongside a dominant profile) or at lower defining proportions than the C40 variant, except in two cases where the C15h/C40‐C15‐C3‐C115 profile was detected (Table S2). Despite their low defining proportions in *Cladocopium* symbiont profiles, these indicator variants served to differentiate regional symbiont assemblages.

**FIGURE 3:**
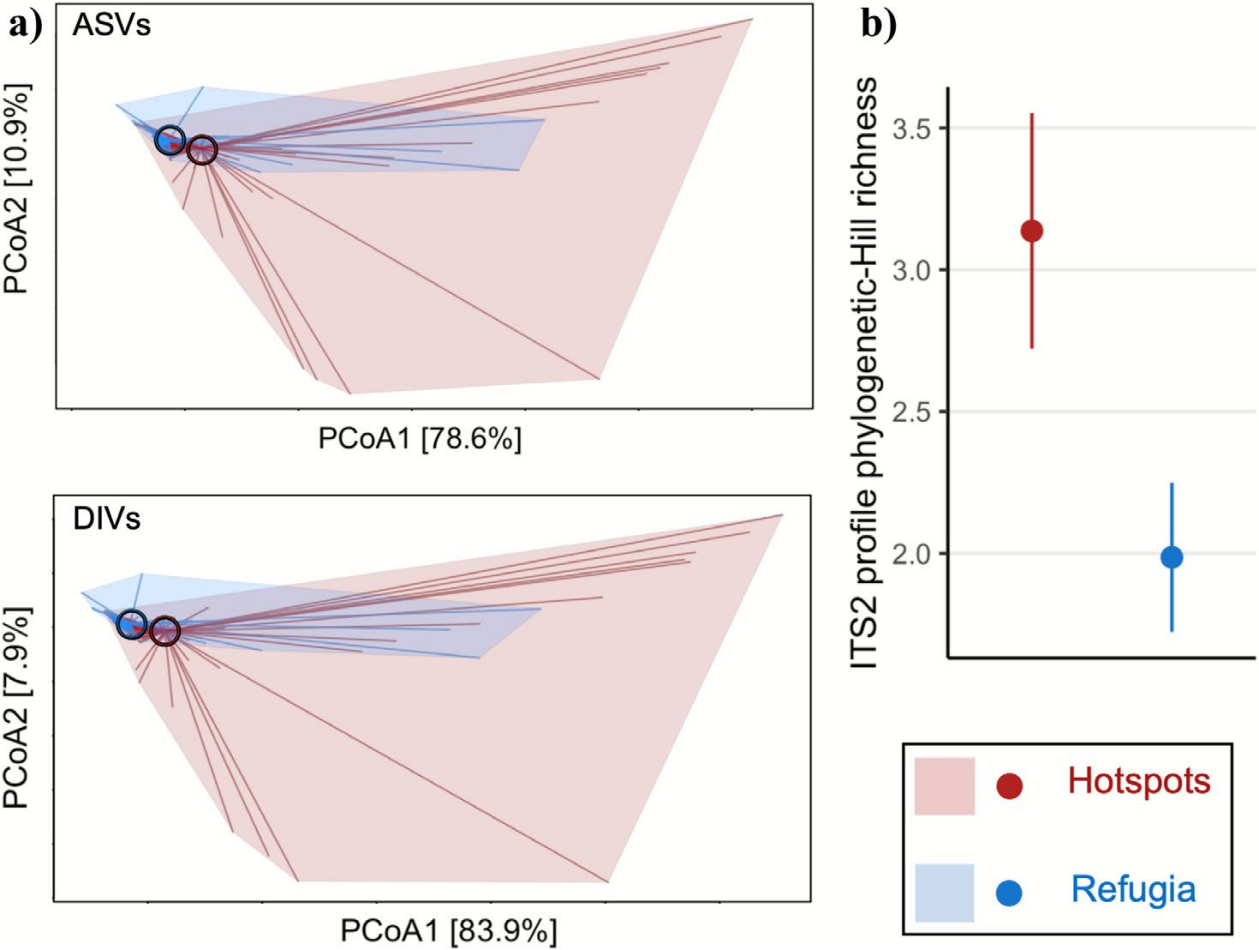
Increased symbiont diversity between and within hotspot corals. (a) Principal coordinate analysis of weighted unifrac distances between symbiont sequence variants. Lines connect the multivariate locations of individual samples to their regional centroids (circled). Shaded red and blue polygons indicate the full multivariate expanse of samples in hotspot and refugium regions respectively. (b) Within‐sample alpha diversity of ITS2 profiles is summarised for each region (hotspot reefs in red, refugium reefs in blue) using phylogenetic‐Hill richness (incorporating richness and summed phylogenetic tree branch lengths, then multiplied by 10^3^ branch units for *y*‐axis interpretability). Points and error bars indicate regional means ± 1 standard error.

Symbiodiniaceae assemblages hosted by corals from hotspot reefs were more heterogeneous than those from thermal refugia, with higher symbiont diversity within and between hotspot corals, quantified as alpha diversity and beta dispersion respectively. A total of 18 distinct *Cladocopium* ITS2 profiles (putative species) were detected in corals sampled from thermal refugia (14 dominant and 4 background profiles, composed of 42 distinct DIVs or 53 ASVs), but similarly, 16 distinct profiles (13 dominant and 3 background, composed of 50 distinct DIVs or 63 ASVs) were detected in hotspot corals. However, the beta‐dispersion between Symbiodiniaceae assemblages was significantly higher between hotspot corals than between refugium corals (ASVs; *F* = 19.08, *p* < 0.001, DIVs; *F* = 21.38, *p* < 0.001), based on sequence variant unifrac‐distance ordinations (Figure 3a). In addition, hotspot corals hosted a higher alpha diversity of symbiont ITS2 profiles than refugium corals based on the richness and phylogenetic spread of profiles (phylogenetic divergence between profiles hosted, or divergence relative to profiles in this dataset if only one profile was present; Figure 3b, *t* = 2.34, *p* = 0.020, Figure S3). The greater symbiont beta dispersion between hotspot corals along with their higher alpha diversity of ITS2 profiles remains evident when restricting analyses to the three hotspot reefs and three refugia sampled for experimental heat stress (ASV dispersion; *F* = 11.54, *p* = 0.001, DIV dispersion; *F* = 12.05, *p* = 0.001, phylogenetic‐Hill richness; *t* = 2.30, *p* = 0.023).

### 3.2 Higher Heat Tolerance Mean and Variance in Thermal Refugia

**FIGURE 4:**
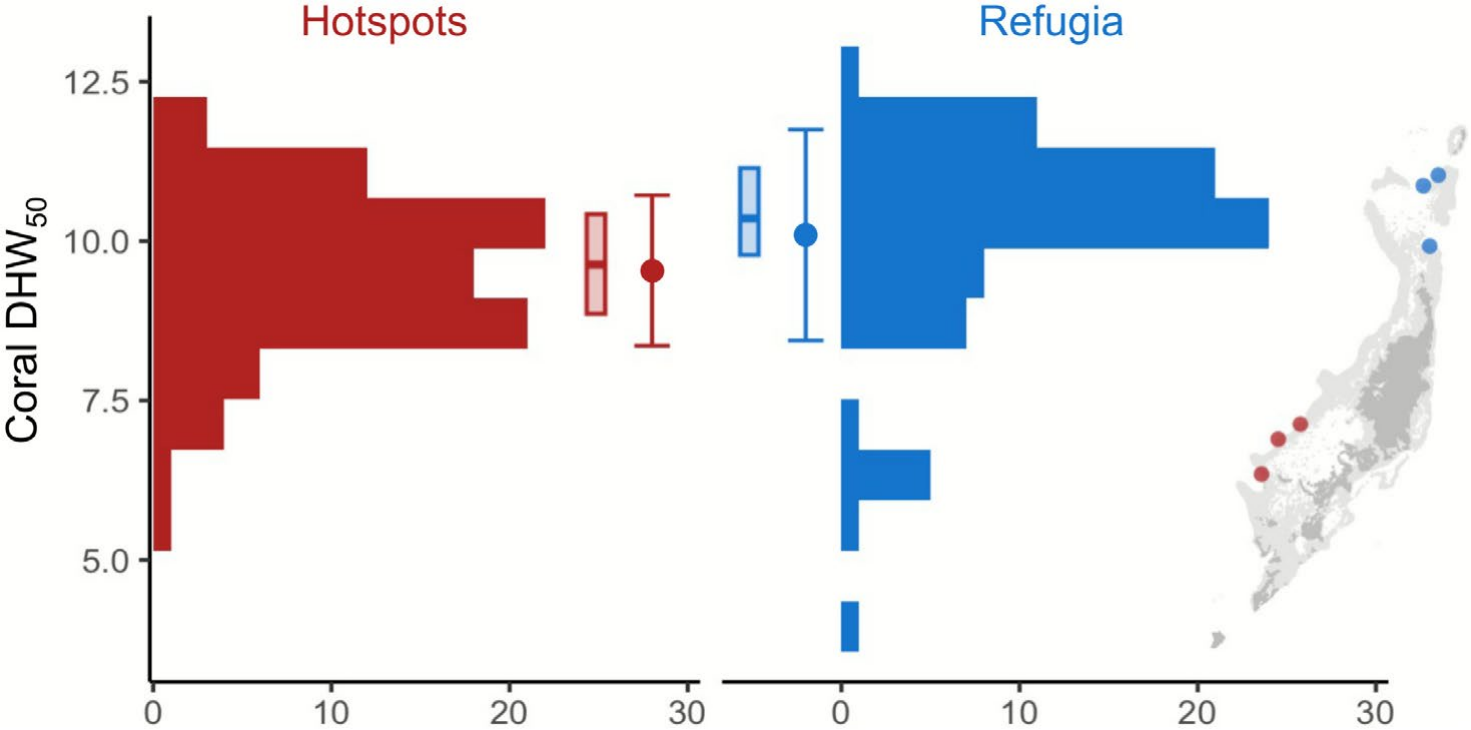
Higher mean, median and variance in heat tolerance among refugium corals. Modified from Lachs, Bozec, et al. (2024); Lachs, Humanes, et al. (2024). Histograms of DHW_50_ for corals collected from three hotspot sites (H1, H2 and H5, *n* = 88) and three thermal refugia (R2, R3 and R4, *n* = 80), marked on map inset. Regional means ± 1 standard deviation are marked by circular points and error bars, whilst boxplots indicate 1st quartile, median and 3rd quartile for each region.

The mean effective dosage of heat stress required to elicit a 50% bleaching mortality response (DHW_50_) was significantly higher for corals from refugia than from hotspot reefs under experimental heat stress (Figure 4; Welch *t* = 2.48, *p* = 0.014; Wilcoxon rank *W* = 2304, *p* < 0.001). This difference represents a mean increased DHW_50_ of 0.54 in refugium corals compared to hotspot corals (refugia mean; 10.09°C‐weeks, hotspots mean; 9.55°C‐weeks). Furthermore, the worst‐ and best‐performing corals under experimental heat stress all originated from thermal refugia (DHW_50_ ranged from 3.8°C to 12.5°C‐weeks), reflecting the higher variance in DHW_50_ between refugium corals compared to hotspot corals (Bartlett's *K* ^2^ = 9.35, *p* = 0.002).

### 3.3 Symbionts With Indicator Variants Linked to Low Heat Tolerance in Hotspot Corals

**FIGURE 5:**
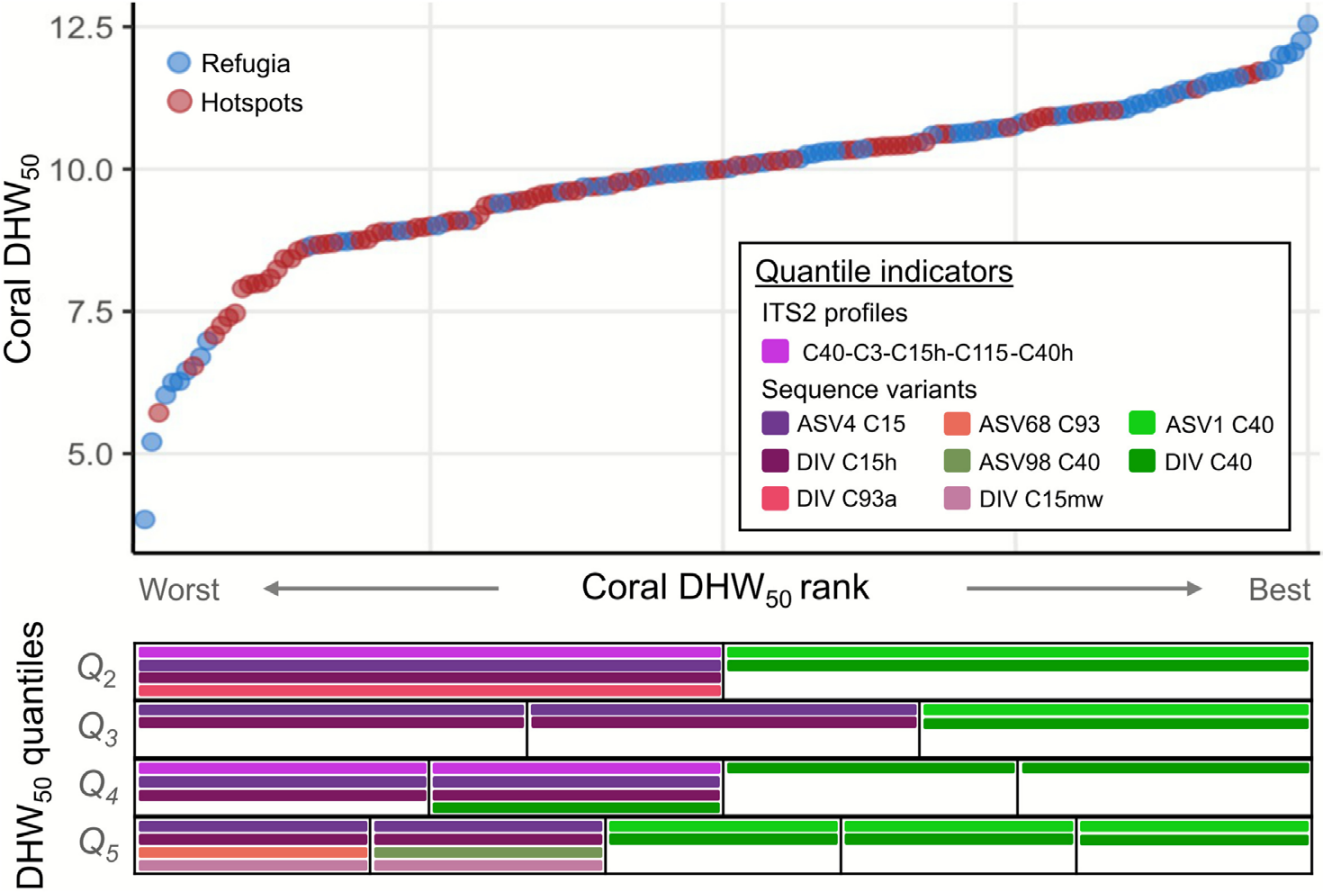
Low‐abundance symbiont sequence variants as low heat tolerance indicators. Corals from the experimental heat stress (*n* = 168) are ranked by increasing DHW_50_ along the *x*‐axis, with blue and red points representing refugium and hotspot corals respectively (top panel). Indicator analyses were performed separately for ASVs, DIVs and ITS2 profiles and were repeated with ranked corals divided into 2, 3, 4 or 5 quantiles (bottom panel). Significant indicators of quantiles are represented by coloured horizontal bars.

Relative read abundances (RRA) of the C40 variant (DIV C40/ASV1 C40), the numerically dominant sequence variant in almost all heat‐stressed corals (mean RRA > 0.8), correlated positively with heat tolerance under experimental heat stress in all four quantile sensitivity analyses (Figure 5, indicator correlation test statistics given in Table S4). Six variants were found to correlate negatively with heat stress performance. Most notable among these are DIV C15h and its close counterpart ASV4 C15 (both with a mean RRA of 0.14 when present), which indicated poor heat stress performance in all four quantile analyses. In addition, ITS2 profile C40‐C3‐C15h‐C115‐C40h, the most common profile of those containing the C15h variant and one with higher occurrence in hotspot corals (Figure 2a), correlated negatively with heat tolerance in two of the four quantile analyses.

**FIGURE 6:**
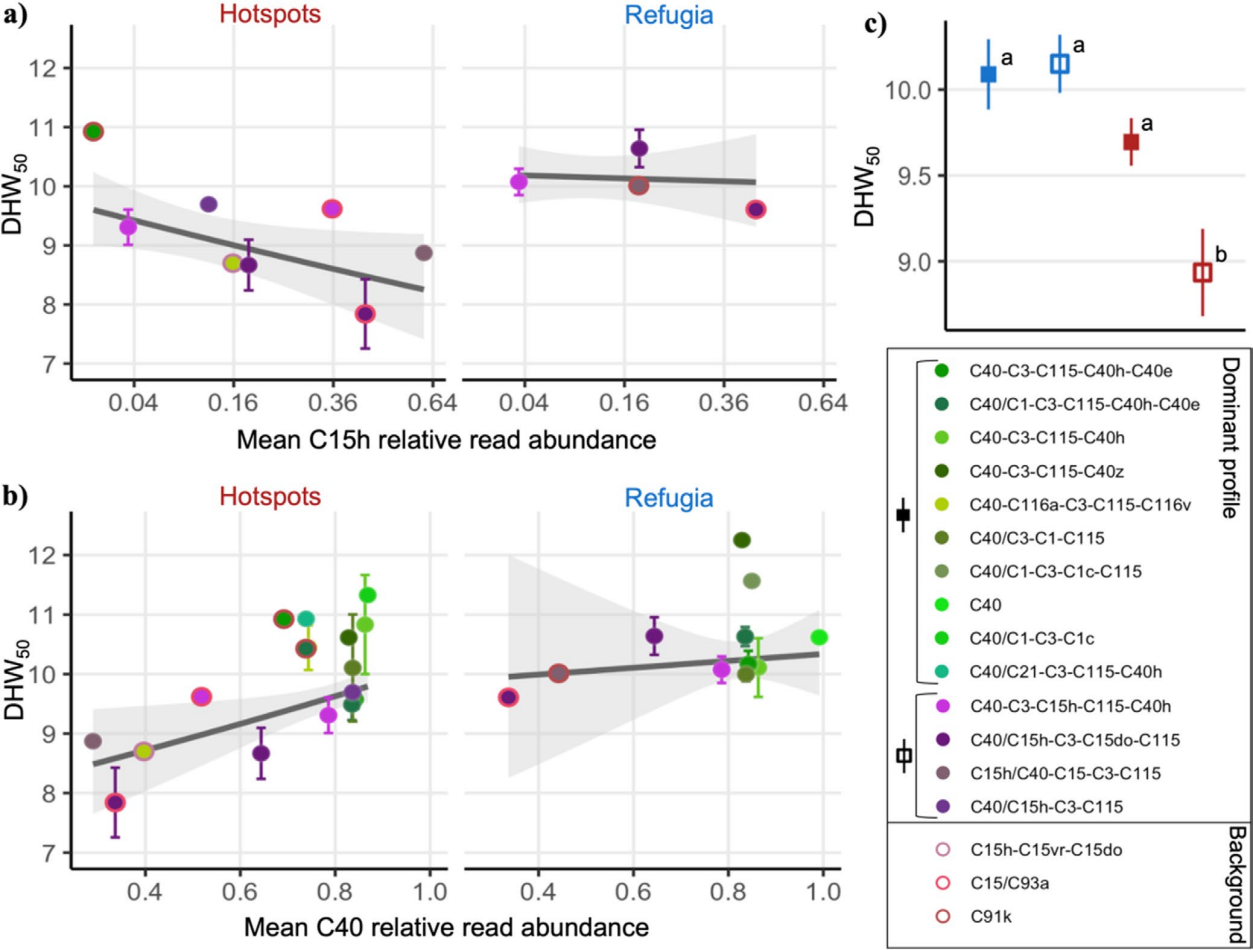
Symbiont indicators only predict heat tolerance at hotspot reefs. (a, b) Points and error bars represent mean DHW_50_ ± one standard error for each unique combination of dominant and background (if present) ITS2 profiles. Generalised linear models with Gamma error distributions are indicated by grey lines, surrounding shading shows ±1 standard error around model predictions. (a) Mean relative read abundance of DIV C15h in profile combinations (*x*‐axis scale is square root transformed). Only profile combinations with non‐zero C15h relative read abundances are shown (hotspots *n* = 19, refugia *n* = 8). (b) Mean relative read abundance of DIV C40 in profile combinations (hotspots *n* = 86, refugia *n* = 78). (c) Mean (±1 standard error) DHW_50_ of refugium (blue) and hotspot (red) corals hosting dominant ITS2 profiles with the C15h variant (empty squares) or without the C15h variant (filled squares). Letter designations represent group differences gleaned from post hoc Tukey's pairwise tests.

The strength of correlations between indicator symbiont candidates and coral heat stress performance was assessed, revealing significant symbiont‐heat tolerance associations for hotspot corals only. The DHW_50_ of hotspot corals decreased significantly with increasing C15h DIV RRA but no analogous effect was detected in corals from thermal refugia, based on corals with non‐zero C15h DIV RRA only (Figure 6a, Hotspots: sample RRA; *z* = 2.26, *p* = 0.024, mean profile RRA; *z* = 2.16, *p* = 0.031, Refugia: sample RRA; *z* = 0.40, *p* = 0.687, mean profile RRA; *z* = 0.26, *p* = 0.795). Additionally, DHW_50_ increased significantly with C40 DIV RRA in hotspot corals but not in refugium corals (Figure 6b, Hotspots: sample RRA; *z* = 3.18, *p* = 0.001, mean profile RRA; *z* = 2.74, *p* = 0.006, Refugia: sample RRA; *z* = 0.39, *p* = 0.697, mean profile RRA; *z* = 0.27, *p* = 0.789). Similarly, hotspot coral DHW_50_ significantly decreased with ASV4 C15 RRA and increased with ASV1 C40 RRA (ASV4: Figure S5a, sample RRA; *z* = 2.80, *p* = 0.005, mean profile RRA; *z* = 2.34, *p* = 0.019, ASV1: Figure S5b, sample RRA; *z* = 2.93, *p* = 0.003, mean profile RRA; *z* = 2.67, *p* = 0.008). In a combined model with both C15h DIV RRA and C40 DIV RRA included as predictors, these symbiotic variables were estimated to explain 10.0%–10.8% of DHW_50_ variation between hotspot corals (sample RRA marginal *R* ^2^; 0.108, mean profile RRA marginal *R* ^2^; 0.100) compared to 0.2%–0.4% of variation between refugium corals (sample RRA marginal *R* ^2^; 0.004, mean profile RRA marginal *R* ^2^; 0.002). The symbiont‐heat tolerance correlations in hotspot corals seem to be largely driven by those hosting ITS2 profiles C40‐C3‐C15h‐C115‐C40h or C40/C15h‐C3‐C15do‐C115 (Figure 6a,b). The mean DHW_50_ for hotspot corals hosting any profile with the C15h variant was only 8.9 compared to the mean DHW_50_ of 9.7 across remaining hotspot corals, equating to these C15h‐containing corals having a lower heat tolerance of 0.8°C‐weeks (Figure 6c).

## 4 Discussion

**FIGURE 7:**
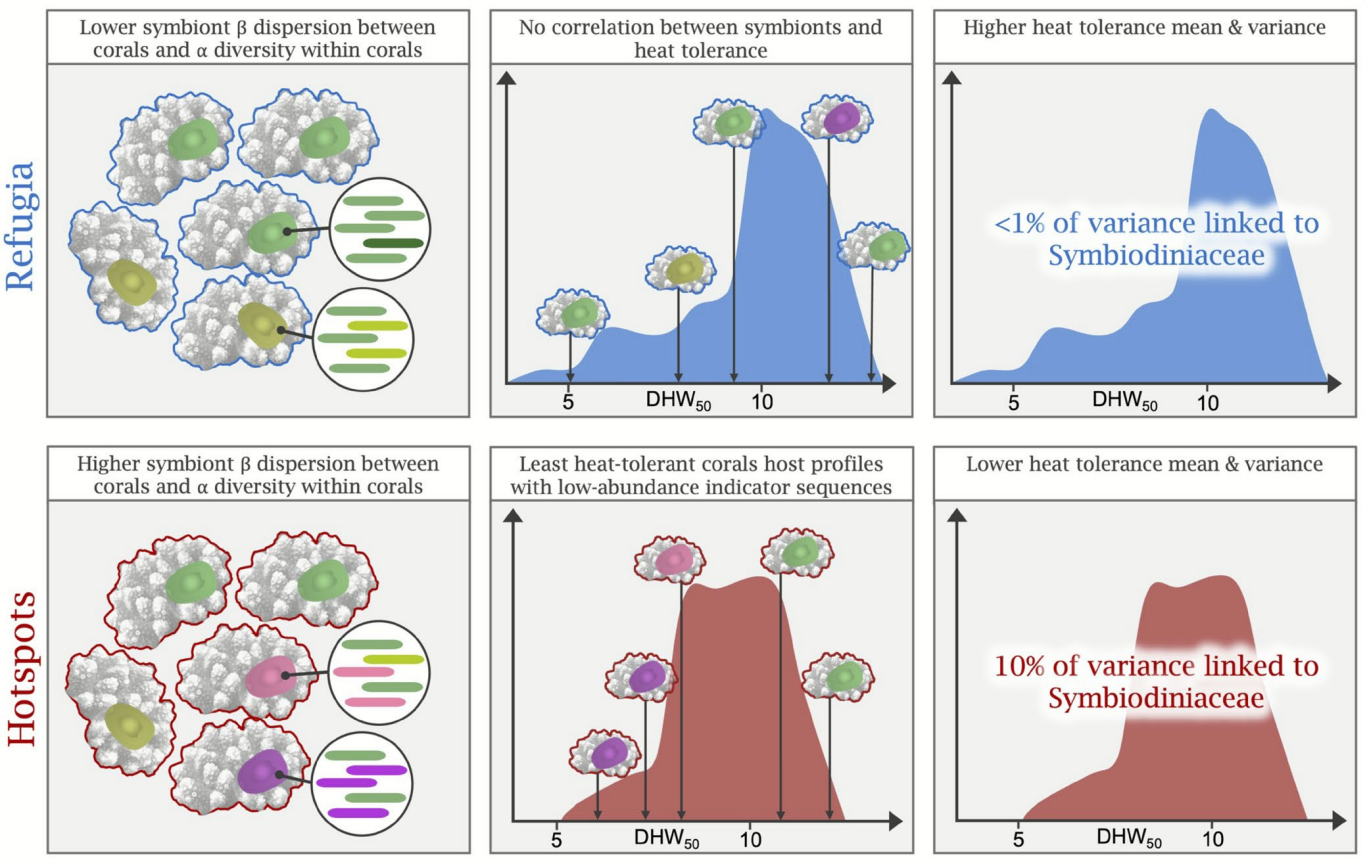
Summary illustration of the region‐dependent distribution of symbiont assemblages containing diverse low‐abundance sequences and their region‐dependent association with low coral heat tolerance. Left panels show 5 *Acropora aff. digitifera* corals representative of thermal refugia (corals outlined in blue) or thermal hotspot reefs (corals outlined in red) identified around Palau. Coloured ovals in corals represent Symbiodiniaceae, with those in refugium corals more similar to each other than those from hotspot corals. Magnified views of symbionts represent the sequence variants detected in symbiont taxa using DNA metabarcoding. Symbionts in hotspot‐corals contained a greater diversity of low‐abundance sequence variants (represented by pink/purple bars) in addition to the C40 variants (shades of green) that dominated this dataset. Middle and right panels show the approximate distributions of coral heat tolerance (DHW_50_) through an experimental heatwave, with a higher mean and variance in heat tolerance for refugium corals. Middle panels are annotated to illustrate the relationship between indicator symbionts (pink/purple) and low heat tolerance among hotspot corals and the lack of association between heat tolerance and symbiont identity among refugium corals.

Using field‐sampling, experimental heatwave exposure, and indicator symbiont analyses, we characterised Symbiodiniaceae assemblages in *Acropora aff. digitifera* across Palauan reefs with different thermal histories and assessed how symbiotic variation relates to coral tolerance to a simulated marine heatwave. We observed a higher diversity of *Cladocopium* symbiont species and their low‐abundance sequence variants in corals from hotspot reefs that had consistently experienced higher levels of heat stress, rather than the symbiont homogenisation or decline in symbiont richness reported in previous studies (Kriefall et al. 2022; Starko et al. 2023; Leiva et al. 2025). Symbiotic differences could not explain the variation in heat tolerance among corals from thermal refugia reported by Lachs, Humanes, et al. (2024), yet symbiont associations could explain some hotspot heat tolerance variability. Together, these results represent a region‐dependent distribution of indicator symbionts coupled with their region‐dependent association with low coral heat tolerance (Figure 7): *Acropora aff. digitifera* from hotspot reefs were more likely to host diverse symbiont species (characterised by their low‐abundance sequence variants) and those hosting such assemblages were more likely to exhibit lower heat stress tolerance compared to other hotspot corals.

### 4.1 Symbiont Diversity Under Chronic Environmental Stress

Symbiont assemblages hosted by *Acropora aff. digitifera* corals from southwestern hotspot forereefs were more diverse and more distinct from each other compared to those hosted by their northeastern conspecifics from thermal refugium forereefs. This difference was largely driven by hotspot corals that hosted *Cladocopium* ITS2 profiles containing small relative abundances of C15 or C116 variants, which were more phylogenetically distinct from the typical C40‐dominated profiles detected in corals in this study. These symbiont differences may reflect differential historical heat stress between these two regions, with thermal hotspots receiving on average an additional 2°C‐weeks during Palau's marine heatwaves in 1998, 2010 and 2017. From 1985 to 2020, hotspots had only 3–4 years without heat stress accumulation, compared to 8–12 heat‐stress free years at thermal refugia (Lachs, Humanes, et al. 2024). However, due to spatial autocorrelation of hotspots and refugia on Palau's southwestern and northeastern forereefs respectively, the effects of other regional environmental variables on *Acropora aff. digitifera*'s symbiont assemblages cannot be overlooked. Specifically, corals in the southern lagoon enclosed by Palau's barrier reef system host Symbiodiniaceae distinct from their outer barrier reef conspecifics (Turnham et al. 2025), linked to the lagoon's comparatively warm, acidic and turbid waters which wash over southern forereefs (including hotspot sites) during receding tides (van Woesik et al. 2012; Golbuu et al. 2016). Although symbiotic differences between hotspot and refugium reefs cannot solely be attributed to historical heat stress, hotspot reefs do characterize increased exposure to chronic environmental stressors compared to their northern refugium counterparts.

Contrary to expectations of environmental filtering of stress‐sensitive symbiont taxa from hotspot reefs, similar numbers of distinct symbiont ITS2 profiles were detected on hotspot and refugium reefs. Evidence of decreased richness or beta dispersion of Symbiodiniaceae taxa in corals, populations or communities comes from a variety of locations in response to recent heatwave exposure (Claar et al. 2020; Jain et al. 2020; Quigley et al. 2022; Starko et al. 2023) or chronic exposure to unfavourable environmental conditions such as high turbidity, salinity, nitrogen, acidity or temperature variability (Fabricius et al. 2004; Ziegler et al. 2017; Smith et al. 2020; Kriefall et al. 2022; Leiva et al. 2025). Whilst we found no evidence to suggest historical heat stress has constrained the diversity of symbionts in the C40 *Cladocopium* lineage found across hotspot reefs, individual corals from Palau's thermal hotspots hosted more diverse and more dissimilar symbiont assemblages. This increased symbiont heterogeneity within and between corals is consistent with the Anna Karenina hypothesis of stress‐induced microbiome dispersion (Zaneveld et al. 2017). Disturbance‐induced increases in richness and heterogeneity are commonly observed in corals' bacterial microbiome (McDevitt‐Irwin et al. 2017), attributed to the vulnerability of health‐compromised hosts to opportunistic microbial colonisation. Here we present an analogous effect of historical heatwave disturbance on the dissimilarity between corals' Symbiodiniaceae assemblages. This result is concordant with evidence of increased richness and dissimilarity of *Cladocopium* lineages hosted by corals from more thermally variable reefs compared to neighbouring reef slopes (van Oppen et al. 2018).

### 4.2 Low‐Abundance Symbiont Variants Associated With Poor Coral Heat Tolerance

*A. aff. digitifera* corals sourced from hotspot reefs that hosted different Symbiodiniaceae taxa displayed significantly different experimental heat tolerances. Differences in heat tolerance among corals could have arisen either because the symbionts themselves differ functionally and directly affect host performance, or because corals with different heat tolerances hosted particular symbiont taxa without a causal link. Considering the increased symbiotic diversity detected in these hotspot corals, a potential mechanism for the latter hypothesis is a reduced host control over symbiont associations in corals weakened by prior heat stress exposure or other environmental stress, which then exhibited low heat tolerance (Carilli et al. 2010; Prada et al. 2017; Baum et al. 2023). Alternatively, the lack of correlation between heat tolerance and symbiont composition in refugium corals may reflect an interaction between chronic environmental stress and symbiont phenotype. Experimental evidence has revealed the degree of energetic mutualism or parasitism exhibited by Symbiodiniaceae *in hospite* to be dynamic in response to environmental change, with direct effects on host physiology (Baker et al. 2018; McIlroy et al. 2020), hence the same Symbiodiniaceae taxon may theoretically confer reduced host benefits to hotspot corals compared to their refugium conspecifics.

The use of the C15h sequence variant as an indicator for low heat tolerance allowed us to distinguish a group of related symbiont taxa (profiles) containing C15h (Figure S3), which were significantly associated with reduced coral heat tolerance. Notably, the difference of 0.8°C‐weeks in mean heat tolerance between hotspot corals hosting these different symbiont profiles is greater than the difference in mean heat tolerance between hotspot and refugium‐sourced *A. aff. digitifera* corals (Lachs, Humanes, et al. 2024). Although these C15h‐containing symbiont profiles largely belong to the C40 lineage of *Cladocopium*, they may still differ functionally. Significant variation in thermotolerance at similarly fine taxonomic scales has previously been reported between taxa within the *Cladocopium* C15 lineage (Hoadley et al. 2021). The C40 variant was also identified as a potential indicator of heat tolerance, although its relative read abundance was often inversely proportional to both that of C15h and to the presence of background ITS2 profiles. This raises the possibility of an emergent property of symbiont assemblages, such as ITS2 profile richness, being associated with holobiont heat tolerance (Kenkel and Bay 2018; Howe‐Kerr et al. 2020). Indeed, despite very low relative read abundances, symbionts hosted at background levels can have disproportionately large effects on holobiont phenotype (Ziegler et al. 2018; Buzzoni et al. 2023).

### 4.3 Non‐Symbiotic Correlates of Coral Heat Tolerance in Thermal Refugia

Experimental heat tolerance of thermal refugium‐sourced corals ranged from 3.8°C to 12.5°C‐weeks, yet differences in the *Cladocopium* symbionts hosted were not linked to heat tolerance differences as they were for hotspot corals. A range of phenotypic differences between coral hosts can influence heat tolerance variability in holobionts (Kenkel et al. 2013; Drury 2020), such as coral tissue biomass, which has been linked to increased heat stress survival in several coral species (Anthony et al. 2009; Thornhill et al. 2011; Huffmyer et al. 2021). The coral tissue biomass of replicate branches from the corals heat‐stressed in this study was reported by Lachs, Humanes, et al. (2024), revealing a correlation between increased tissue biomass and heat tolerance in refugium corals, with no discernible relationship in hotspot corals. Variation in tissue biomass could explain 5.3% of heat tolerance variation between refugium corals (Figure S6), which when considered alongside the 10% of variation explained by Symbiodiniaceae in hotspot corals reported here, leaves a large amount of unexplained heat tolerance variation in *Acropora aff. digitifera* corals from both regions. Some of this remaining variability may be linked to the additive genetic variance between hosts: the narrow‐sense heritability of heat tolerance in *A. aff. digitifera* corals from the southwest of Palau's reef system has previously been estimated as 0.23 (23% of variation explained), following the same experimental heat stress procedure reported here (Humanes et al. 2024). The region‐specific associations of symbiont assemblage and tissue biomass with heat tolerance in these *A. aff. digitifera* corals highlight the potentially transformative effect of local environmental history on the relationship between host, Symbiodiniaceae and heatwave performance (Howells et al. 2012; Drury and Lirman 2021; Naugle et al. 2021), even within a single coral population.

As the world's coral reefs face intensifying marine heatwave exposure under accelerating climate change (Oliver et al. 2021; Cooley et al. 2022), we reveal aspects of coral‐Symbiodiniaceae ecology likely to become increasingly relevant for the persistence of these ecosystems. Namely, coral populations that consistently host symbionts within one ancestral lineage of genus *Cladocopium* may still show a degree of symbiotic flexibility in response to environmental change. Furthermore, symbiont differences at these fine taxonomic scales can be associated, whether causal or correlational, with definitive differences in coral resilience to marine heatwaves. These findings are applicable to symbiont‐informed predictions of heatwave vulnerability and in mapping the distribution of ‘at‐risk’ reefs across these small spatial scales (Fuller et al. 2020), a useful tool in the prioritisation of reef management efforts (McLeod et al. 2021). Furthermore, our results demonstrate the potential for using symbiont metabarcoding to detect holobionts with low heat tolerance within a population, even in coral taxa associated with low Symbiodiniaceae diversity. Similar approaches could be incorporated into reef restoration to screen for potentially maladaptive indicator symbionts, thereby avoiding propagating them through coral nurseries and onto restored reefs (Baums et al. 2019).

## Author Contributions

Conceptualisation: D.B., L.L., J.B., A.J.E., Y.G., A.H., H.M.M., G.M., J.K.B. and J.R.G. Data collection: D.B., L.L., E.B., L.B., J.B., A.H., H.M.M., G.M. and J.R.G. Data processing: D.B., L.L., E.B., L.B., J.B., A.J.E., A.H., H.M.M. and J.R.G. Data analysis: D.B. and L.L. Writing (original draft): D.B. Writing (review and editing): D.B., L.L., E.B., L.B., J.B., A.J.E., Y.G., A.H., H.M.M., G.M., J.K.B. and J.R.G.

## Funding

This work was supported by The Leverhulme Trust, SAS‐2021‐047; Government of Canada's New Frontiers in Research Fund, NFRFT‐2020‐00073‐BIOSCAN; International Coral Reef Society; HORIZON EUROPE European Research Council, 725848.

## Disclosure

Research Permits for visiting and collecting coral branches were approved by Palau’s national government (Ministry of Natural Resources, Environment, and Tourism: RE‐22‐11) and by the individual States visited (Ngarchelong State, Kayangel State, and Koror State). All work was in accordance with the ethical standards of both Newcastle University and the University of Victoria. This research contributes to a long‐standing collaboration between the Palau International Coral Reef Center and Newcastle University, which in‐part aims to understand the basis of heat‐tolerance variation in coral and how this can be leveraged to restore and improve the persistence of Palau’s coral reefs under climate change.

## Conflicts of Interest

The authors declare no conflicts of interest.

## Supporting information


**Appendix S1:** mec70243‐sup‐0001‐AppendixS1.docx.

## Data Availability

Raw ITS2 DNA metabarcoding sequence data are available on the NCBI Sequence Read Archive under the BioProject accession 1295668. All other data and code are available on GitHub (https://github.com/DaisyBuzzoni/Buzzoni_etal_2025_Palau_Adigitifera_symbionts) and archived at DOI:10.5281/zenodo.18249107.
