# Supplementary material for: Algal Symbionts Indicate Heatwave Vulnerability in Corals From Hotspots but Not From Thermal Refugia
Source: Mol Ecol. 2026 Jan 17;35(2):e70243. doi: 10.1111/mec.70243 (PMC12811821; doi:10.1111/mec.70243)
Supplement: Supplementary file 1 — Appendix S1: mec70243‐sup‐0001‐AppendixS1.docx. [file MEC-35-e70243-s001.docx]

**Supplemental Materials**

**Algal symbionts indicate heatwave vulnerability in corals from hotspots but not from thermal refugia**

Daisy Buzzoni, Liam Lachs, Elizabeth Beauchamp, Leah Bukurou, John Bythell, Alasdair J. Edwards, Yimnang Golbuu, Adriana Humanes, Helios M. Martinez, Geory Mereb, Julia K. Baum, James R. Guest.

| **Site** | **Latitude** | **Longitude** | **Collection date** | **Branches in heat stress** | **Colonies in heat stress** | **Colonies sequenced** |
| --- | --- | --- | --- | --- | --- | --- |
| Hotspot 1 (H1) | 7.409 | 134.332 | 05-Apr | 143 | 29 | 29 |
| Hotspot 2 (H2) | 7.374 | 134.280 | 04-Apr | 145 | 29 | 29 |
| Hotspot 3 (H3) | 7.334 | 134.245 | 15-May | 0 | 0 | 29 |
| Hotspot 4 (H4) | 7.324 | 134.218 | 16-May | 0 | 0 | 30 |
| Hotspot 5 (H5) | 7.292 | 134.240 | 05-Apr | 148 | 30 | 30 |
| Refugium 1 (R1) | 8.006 | 134.681 | 20-May | 0 | 0 | 29 |
| Refugium 2 (R2) | 7.989 | 134.659 | 03-Apr | 138 | 28 | 29 |
| Refugium 3 (R3) | 7.965 | 134.624 | 03-Apr | 102 | 24 | 30 |
| Refugium 4 (R4) | 7.824 | 134.639 | 03-Apr | 140 | 28 | 28 |
| Refugium 5 (R5) | 7.818 | 134.6580 | 19-May | 0 | 0 | 30 |
| Total | | | | 816 | 168 | 293 |

**Table S1: Coral collection sample sizes and site metadata.** Sample sizes represent those used in analyses, following sample sequence quality control and the removal of colonies with unexplained control mortality in experimental heatwave.

| **ITS2 profile (dataset detections)** | **Average defining sequence proportions and [stdev]** |
| --- | --- |
| C40 (1) | 1.000[NA] |
| C40/C1-C3-C1c (1) | 0.911[NA]-0.054[NA]-0.026[NA]-0.009[NA] |
| C40-C3-C115-C40h (16) | 0.895[0.030]-0.058[0.018]-0.037[0.019]-0.010[0.002] |
| C40-C3-C115-C40h-C40e (173) | 0.871[0.029]-0.061[0.015]-0.040[0.010]-0.019[0.006]-0.009[0.004] |
| C40/C1-C3-C1c-C115 (4) | 0.872[0.029]-0.055[0.019]-0.036[0.012]-0.029[0.016]-0.008[0.005] |
| C40/C3-C1-C115 (6) | 0.870[0.023]-0.071[0.022]-0.035[0.010]-0.024[0.015] |
| C40-C3-C115-C40z (8) | 0.863[0.040]-0.073[0.019]-0.048[0.022]-0.017[0.004] |
| C40-C40i-C3-C115 (2) | 0.862[0.026]-0.062[0.007]-0.039[0.016]-0.037[0.003] |
| C40/C1-C3-C115-C40h-C40e (27) | 0.858[0.031]-0.068[0.018]-0.042[0.013]-0.015[0.005]-0.011[0.013]-0.007[0.002] |
| C40-C40i-C3-C40h-C40e (2) | 0.775[0.008]-0.144[0.006]-0.053[0.005]-0.017[0.002]-0.011[0.001] |
| C40/C21-C3-C115-C40h (1) | 0.777[NA]-0.116[NA]-0.061[NA]-0.031[NA]-0.015[NA] |
| C40/C21 (1) | 0.500[NA]-0.500[NA] |
| C40-C116a-C3-C115-C116v (7) | 0.729[0.116]-0.144[0.099]-0.057[0.014]-0.036[0.007]-0.035[0.026] |
| C40-C3-C15h-C115-C40h (32) | 0.830[0.067]-0.060[0.018]-0.053[0.066]-0.040[0.013]-0.017[0.006] |
| C40/C15h-C3-C115 (1) | 0.856[NA]-0.125[NA]-0.011[NA]-0.009[NA] |
| C40/C15h-C3-C15do-C115 (11) | 0.532[0.199]/0.375[0.199]-0.041[0.013]-0.029[0.012]-0.023[0.008] |
| C40/C15h-C40i-C3 (1) | 0.468[NA]-0.462[NA]-0.044[NA]-0.027[NA] |
| C15h/C40-C15-C3-C115 (2) | 0.467[0.238]/0.447[0.259]-0.043[0.010]-0.029[0.009]-0.015[0.002] |
| C15h-C15vr-C15do (1) | 0.843[NA]-0.104[NA]-0.054[NA] |
| C15/C93a (6) | 0.633[0.096]/0.367[0.096] |
| C91k (4) | 1.000[0.000] |
| C50c/C50a-C3-C3b-C50f-C50u (1) | 0.500[NA]-0.380[NA]-0.065[NA]-0.035[NA]-0.016[NA]-0.004[NA] |
| C50c/C50a (1) | 0.541[NA]-0.459[NA] |

**Table S2: ITS2 profile within-dataset mean defining sequence proportions and their standard deviations.**

| **Sequence variant** | **Region indicated** | **Test statistic (IndVal)** | ***P*(IndVal\|H_0_)** |
| --- | --- | --- | --- |
| ASV19 C40 | Hotspots | 0.44 | <0.001 |
| ASV4 C15 | Hotspots | 0.42 | 0.010 |
| ASV14 C116 | Hotspots | 0.22 | 0.014 |
| ASV75 C116 | Hotspots | 0.22 | 0.014 |
| ASV51 C116 | Hotspots | 0.20 | 0.027 |
| ASV40 C116 | Hotspots | 0.20 | 0.027 |
| ASV13 C40 | Refugia | 0.49 | <0.001 |
| ASV87 C115 | Refugia | 0.22 | 0.007 |
| ASV86 C115 | Refugia | 0.20 | 0.015 |
| ASV7 C40 | Refugia | 0.19 | 0.030 |
| ASV95 C40 | Refugia | 0.19 | 0.030 |
| ASV60 C30 | Refugia | 0.19 | 0.031 |
| DIV C40z | Hotspots | 0.73 | <0.001 |
| DIV C1 | Hotspots | 0.41 | 0.002 |
| DIV C15h | Hotspots | 0.41 | 0.003 |
| DIV C116v | Hotspots | 0.22 | 0.015 |
| DIV C116a | Hotspots | 0.22 | 0.015 |
| DIV C116x | Hotspots | 0.20 | 0.028 |
| DIV C15hg | Hotspots | 0.20 | 0.031 |
| DIV C40i | Refugia | 0.19 | 0.030 |

**Table S3: Statistical test results for regional indicator analyses**

| **Profile/variant** | **Test iteration (*Q*_n_)** | **Quantile(s) indicated** | **Test statistic (r)** | ***P*(r\|H_0_)** |
| --- | --- | --- | --- | --- |
| C40-C3-C15h-C115-C40h | *Q*_2_ | *Q*_2_ | 0.20 | 0.018 |
| C40-C3-C15h-C115-C40h | *Q_4_* | *Q_4_ Q_3_* | 0.20 | 0.042 |
| ASV4 C15 | *Q*_2_ | *Q*_2_ | 0.21 | 0.006 |
| ASV4 C15 | *Q_3_* | *Q_3_ Q_2_* | 0.20 | 0.022 |
| ASV4 C15 | *Q_4_* | *Q_4_ Q_3_* | 0.21 | 0.032 |
| ASV4 C15 | *Q_5_* | *Q_5_ Q_4_* | 0.24 | 0.020 |
| DIV C15h | *Q*_2_ | *Q*_2_ | 0.21 | 0.004 |
| DIV C15h | *Q_3_* | *Q_3_ Q_2_* | 0.21 | 0.023 |
| DIV C15h | *Q_4_* | *Q_4_ Q_3_* | 0.21 | 0.029 |
| DIV C15h | *Q_5_* | *Q_5_ Q_4_* | 0.25 | 0.013 |
| DIV C93a | *Q*_2_ | *Q*_2_ | 0.15 | 0.050 |
| DIV C15mw | *Q_5_* | *Q_5_ Q_4_* | 0.21 | 0.035 |
| ASV68 C93 | *Q_5_* | *Q_5_* | 0.27 | 0.015 |
| ASV98 C40 | *Q_5_* | *Q_4_* | 0.22 | 0.030 |
| ASV1 C40 | *Q*_2_ | *Q_1_* | 0.18 | 0.018 |
| ASV1 C40 | *Q_3_* | *Q_1_* | 0.20 | 0.017 |
| ASV1 C40 | *Q_5_* | *Q_1_ Q_2_ Q_3_* | 0.23 | 0.031 |
| DIV C40 | *Q*_2_ | *Q_1_* | 0.21 | 0.004 |
| DIV C40 | *Q_3_* | *Q_1_* | 0.20 | 0.017 |
| DIV C40 | *Q_4_* | *Q_1_ Q_2_ Q_3_* | 0.23 | 0.012 |
| DIV C40 | *Q_5_* | *Q_1_ Q_2_ Q_3_* | 0.25 | 0.016 |

**Table S4: Statistical test results for DHW_50_ quantile indicator analyses**

**
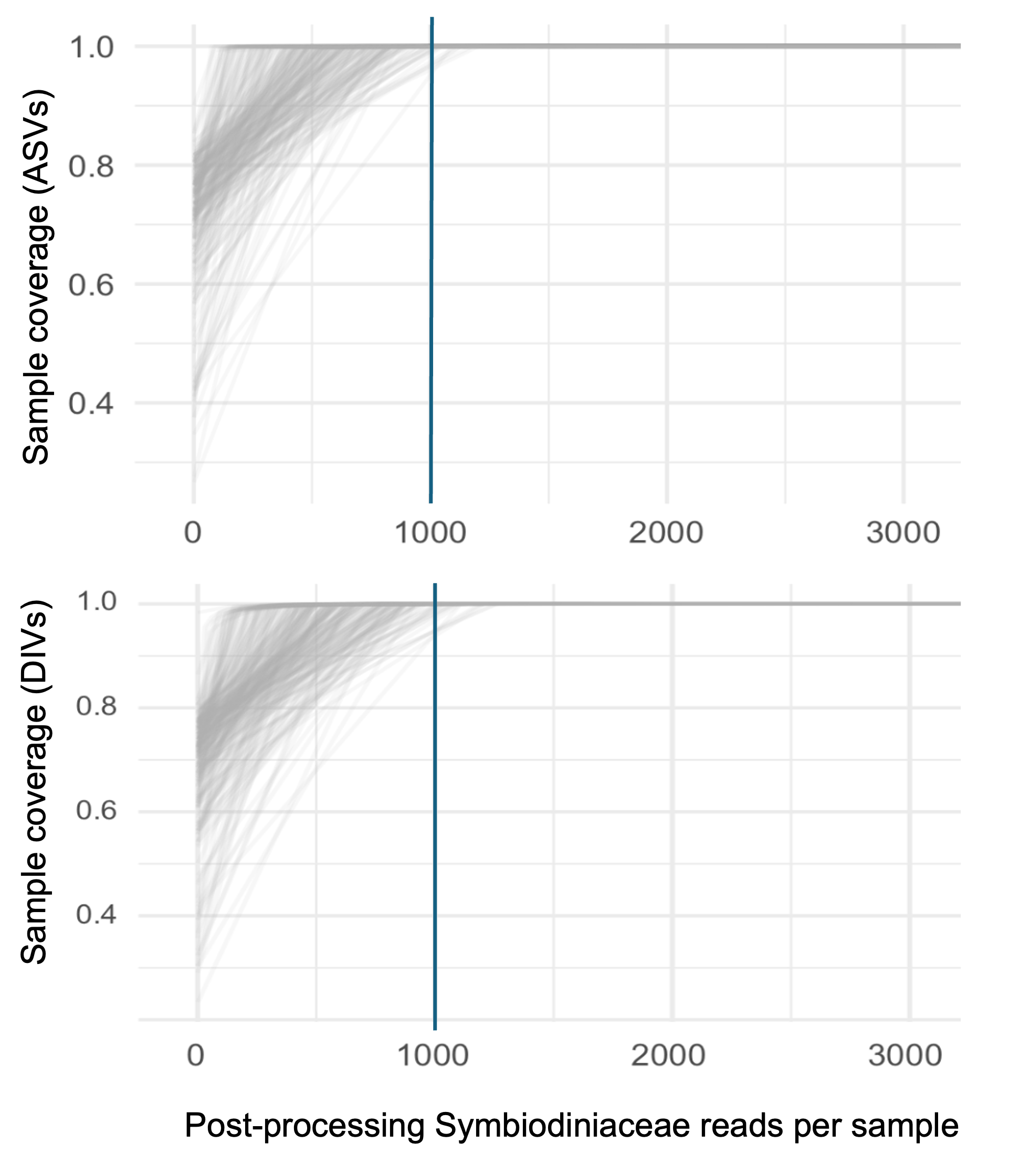
**

**Figure S1:** **Estimated sequence variant coverage informs sample minimum read threshold.** Per-sample estimated coverage of true symbiont sequence richness of ASVs (top) and DIVs (bottom). Each grey line represents a single sample.


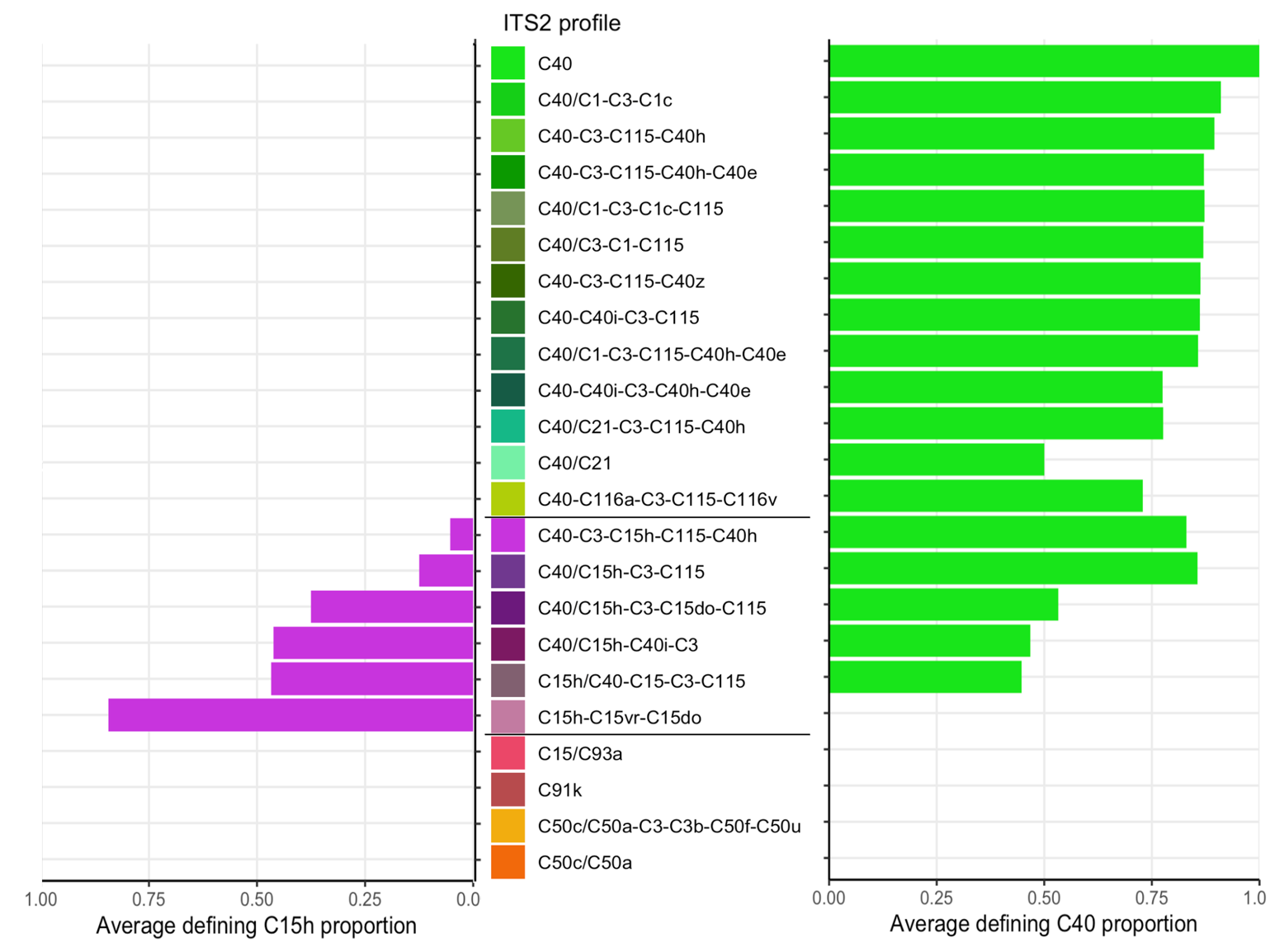


**Figure S2:** **Variant average defining proportions inform ITS2 profile colour scale.** ITS2 profiles can most simply be delineated by the average defining proportions (mean relative read abundances) of sequence variants comprising profile detections within this dataset. Specifically, defining proportions of the C40 (left) and C15h (right) sequence variants have been used to inform the colour-scheme used for profiles due to their high relative read abundances and commonness across samples. Profiles comprising mainly the C40 variant are shown along a green colour-scale (top group) unless the C15h variant is present, in which cases profiles are indicated by purple hues (middle group), followed by profiles comprising neither variant (bottom group).

**
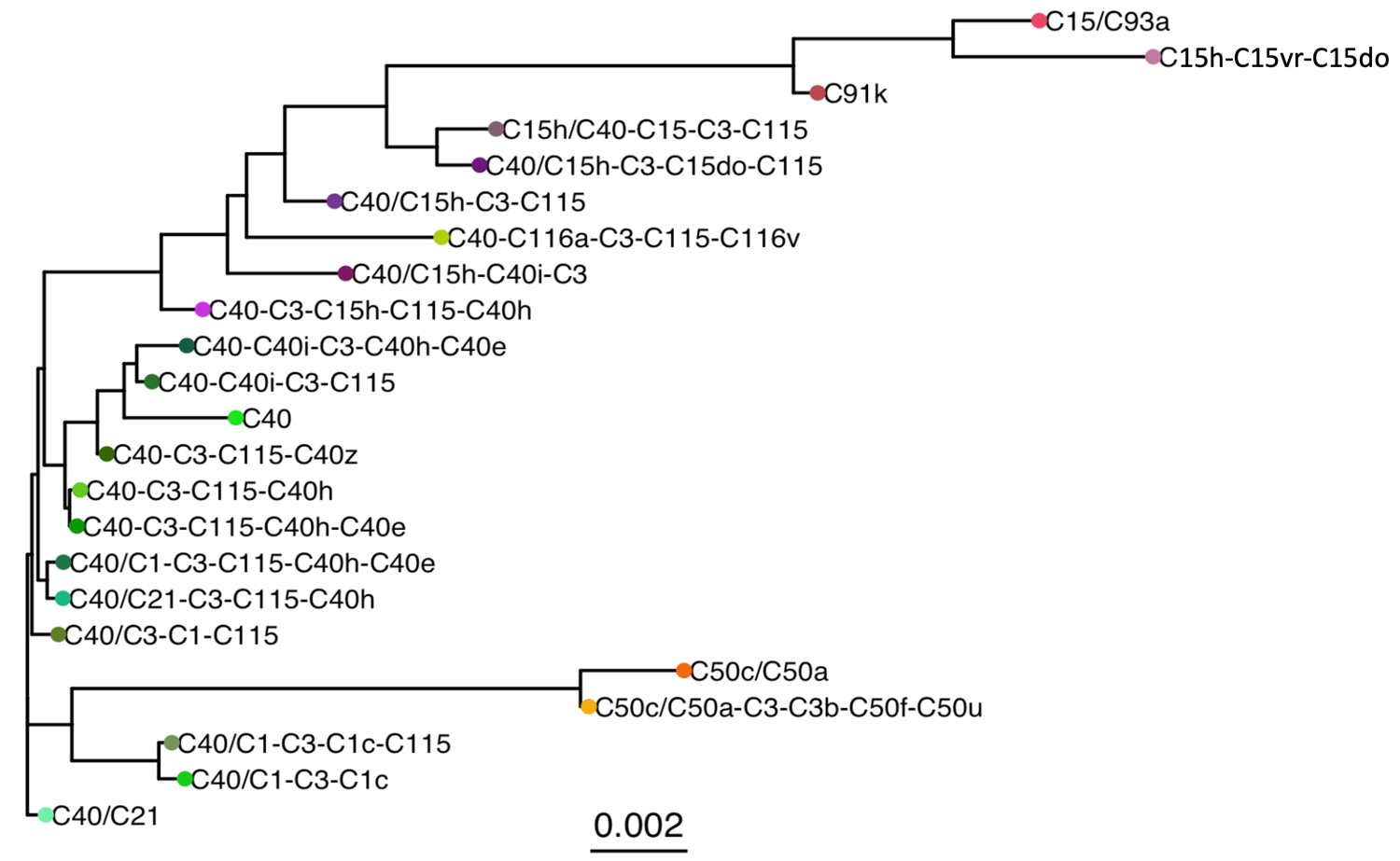
Figure S3:** **Neighbour-joining tree of between-ITS2 profile distances.** Tree built using square root unifrac distances between ITS profiles, provided by SymPortal. Branch lengths are scaled to represent distances, with scale provided below tree.


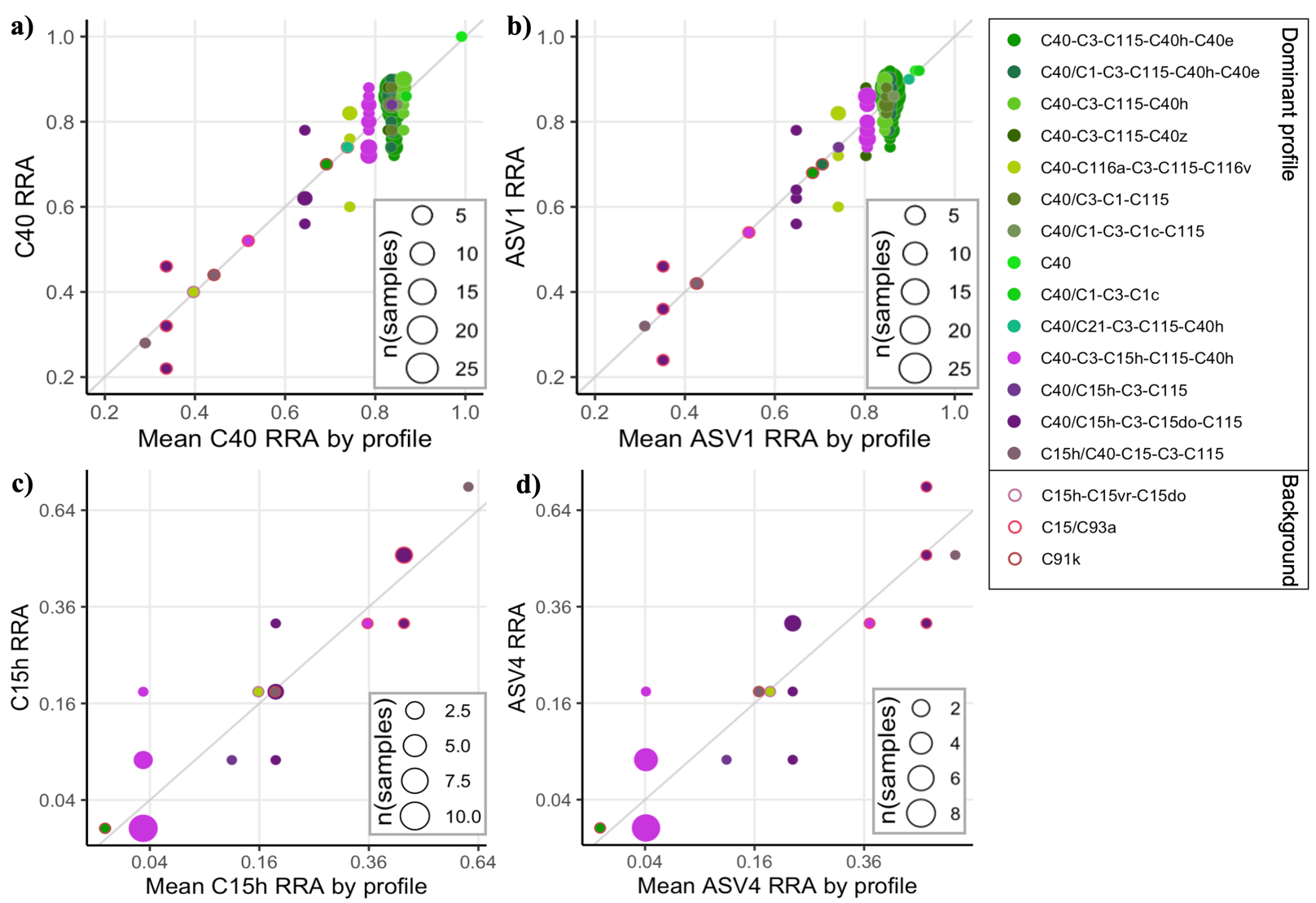
**Figure S4:** **Variability in sample RRA within mean profile RRA for four variants of interest.** Within-sample relative read abundance (RRA) of variants is shown on y-axes, binned into increments of 0.02 RRA with point size representing sample counts. Samples are also grouped by the ITS2 profiles hosted, indicated by point fill (dominant profile) and border colour (background profile, if present). X-axes show the mean RRA for samples within each profile group. **a)** RRA of DIV C40 (n = 164). **b)** RRA of ASV1 C40 (n = 164). **c)** RRA of DIV C15h, only including profile groups containing C15h (axes scales are square root transformed, n = 27). **d)** RRA of ASV4 C15, only including profile groups containing C15h (axes scales are square root transformed, n = 27).

**
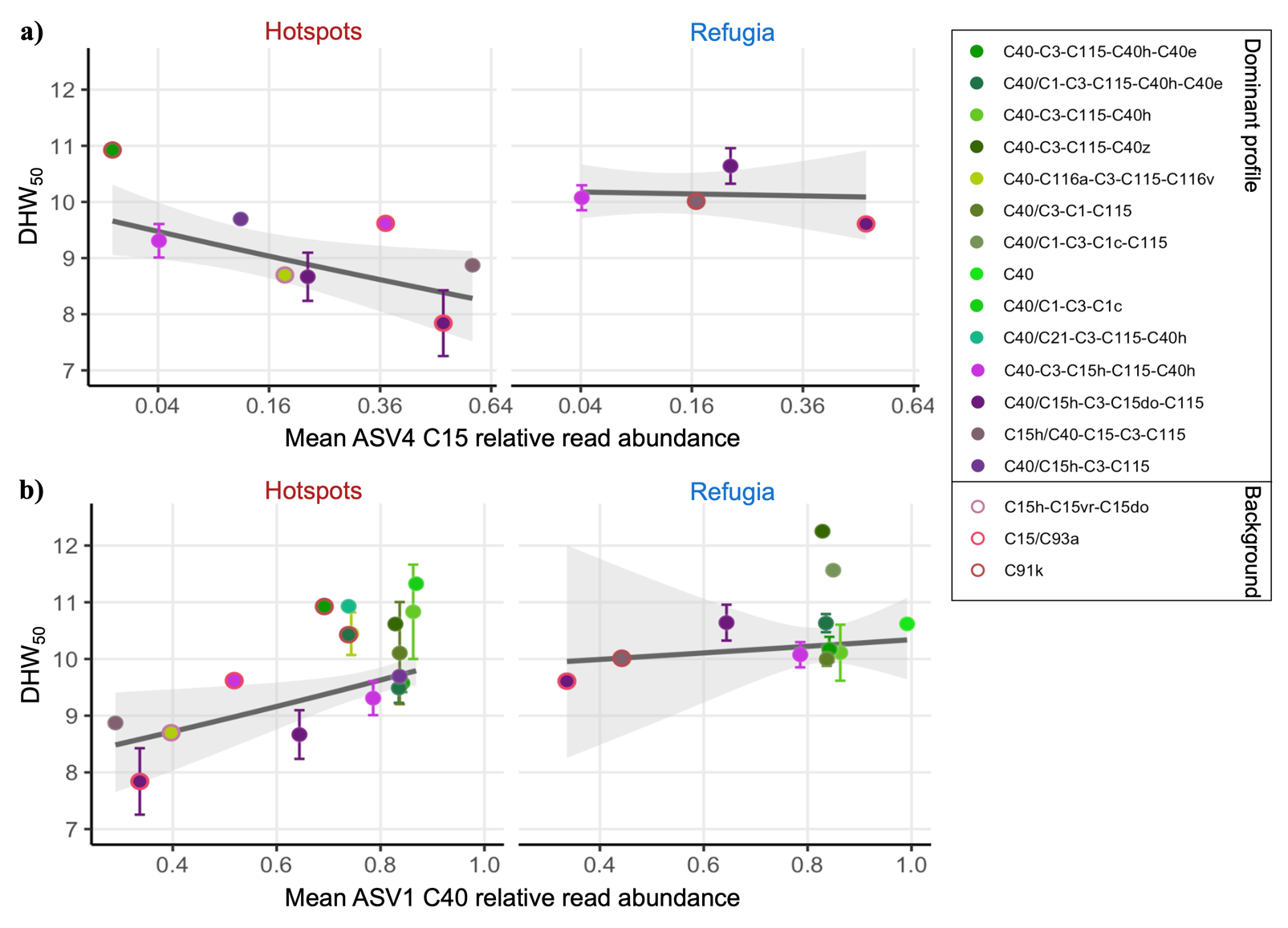
Figure S5:** **Symbiont indicators only predict heat tolerance at hotspot reefs.** Points and error bars represent mean DHW50 +/- one standard error for each unique combination of dominant and background (if present) ITS2 profiles. Generalised linear models with Gamma error distributions are indicated by grey lines, surrounding shading shows +/- 1 standard error around model predictions. **a)** Mean relative read abundance of ASV4 C15 in profile combinations (X-axis scale is square root transformed). Only profile combinations with non-zero ASV4 relative read abundances are shown (hotspots n = 19, refugia n = 8). **b)** Mean relative read abundance of ASV1 in profile combinations (hotspots n = 86, refugia n = 78).


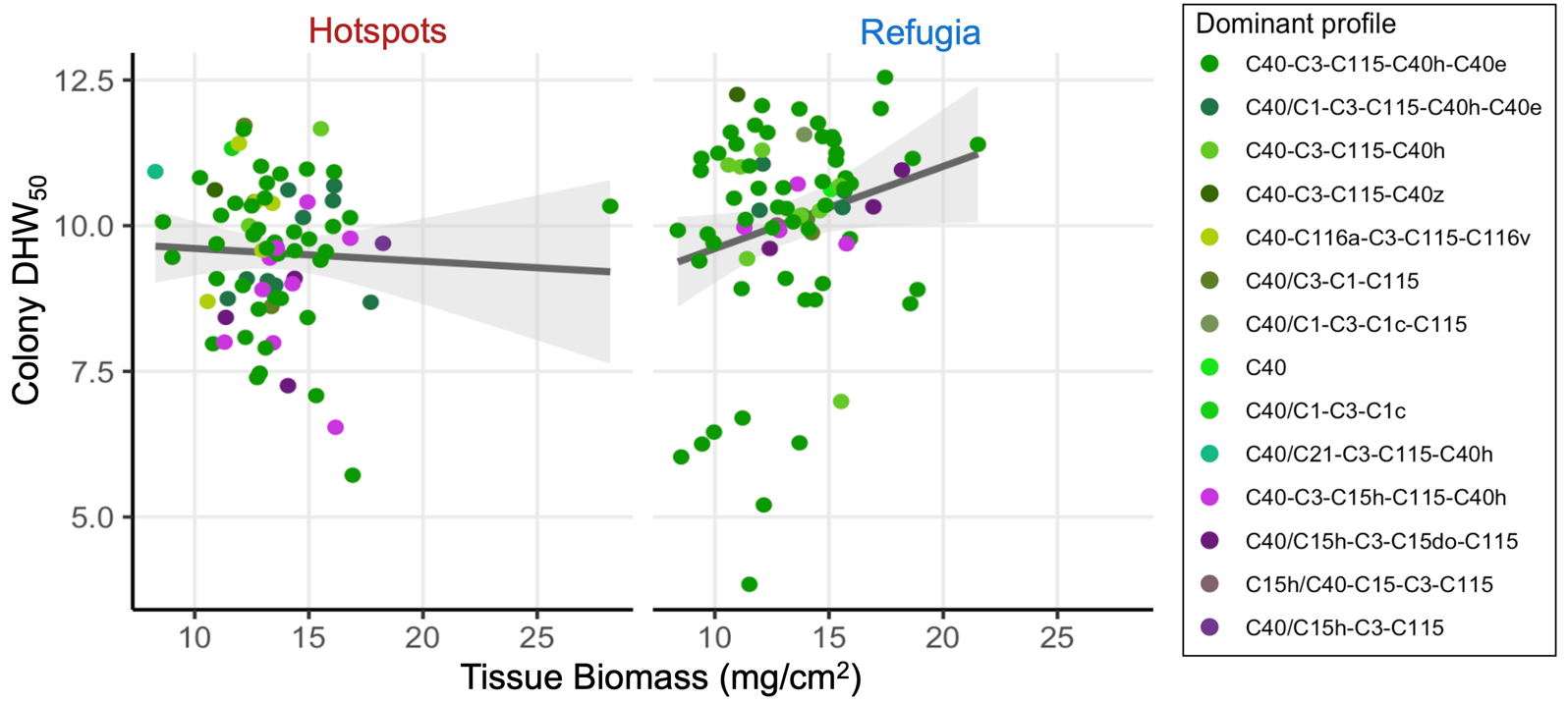
**Figure S6:** **Non-symbiotic correlates of heat tolerance at refugium reefs.** Modified from Lachs et al. (2024). Each point represents a coral colony, coloured by Symbiodiniaceae dominant ITS2 profile. Generalised linear models are indicated by grey lines, surrounding shading shows +/- 1 standard error around model predictions. The mean tissue biomass (from 3 replicate branches) of colonies is shown against colony DHW_50_. A generalised linear mixed effects models (with site included as a random effect) revealed a significant positive effect of tissue biomass on DHW_50_ in refugia (*z* = 2.49, *P* = 0.013), with no significant effect in hotspot corals (*z* = 0.59, *P* = 0.555). Tissue biomass explained an additional 5.3% of heat tolerance variation between refugium corals (marginal R^2^; 0.053) when included in symbiont generalised mixed effects models.
